# The efficacy of self-exercise in a patient with cervicogenic dizziness: A randomized controlled trial

**DOI:** 10.3389/fneur.2023.1121101

**Published:** 2023-02-14

**Authors:** Patorn Piromchai, Nattaporn Toumjaidee, Somchai Srirompotong, Kwanchanok Yimtae

**Affiliations:** Department of Otorhinolaryngology, Faculty of Medicine, Khon Kaen University, Khon Kaen, Thailand

**Keywords:** exercise, vertigo, peripheral vertigo, self-exercise, dizziness

## Abstract

**Background:**

Cervicogenic dizziness is a clinical syndrome characterized by neck pain and dizziness. Recent evidence suggested that self-exercise could improve a patient's symptoms. The objective of this study was to evaluate the efficacy of self-exercise as an add-on therapy in patients with non-traumatic cervicogenic dizziness.

**Methods:**

Patients with non-traumatic cervicogenic dizziness were randomly assigned to the self-exercise and control groups. The self-exercise group was instructed to perform muscle, mobilization, and oculomotor training at home while there was no specific training given to the control group. The neck pain, dizziness symptoms, and their impact on daily life were evaluated by the Dizziness Handicap Inventory (DHI) scale, the Neck Disability Index (NDI) scale, and the visual analog scale (VAS). The objective outcomes included the range of motion test of the neck and the posturography test. All outcomes were evaluated at 2 weeks after the initial treatment.

**Results:**

A total of 32 patients participated in this study. The average age of the participants was 48 years. The DHI score of the self-exercise group after the treatment was significantly lower when compared to the control group [mean difference (MD) 25.92 points, 95% CI 4.21–47.63, *p* = 0.021]. The NDI score after treatment was also significantly lower in the self-exercise group (MD 6.16 points, 95% CI 0.42–11.88, *p* = 0.036). However, there was no statistical difference in the VAS score, the range of motion test, and the posturography test between the two groups (*p* > 0.05). No significant side effects were noted in either of the groups.

**Conclusion:**

Self-exercise is effective in reducing dizziness symptoms and its impact on daily life in patients with non-traumatic cervicogenic dizziness.

## 1. Introduction

Cervicogenic dizziness is a clinical syndrome characterized by neck pain and dizziness. It accounted for ~5% of all dizziness and vertigo encounters in our setting in a previous study (1, 2). In the retrospective chart review, non-traumatic cervicogenic dizziness was found in ENT clinics from 5.42 to 7.5% (3, 4). The prevalence of cervicogenic dizziness from a prospective multicenter study was 6.4% (5).

Cervicogenic dizziness is defined as dizziness from cervical spine disorders (6). The pathophysiology of this disease was from proprioceptive system abnormality (7, 8). Proprioceptive input from the neck helps in the coordination of eye, head, and body posture, as well as spatial orientation (7).

The treatment regimens for non-traumatic cervicogenic dizziness include muscle relaxants, analgesics, and antidizziness drugs. Physiotherapy and manual therapy are also recommended to improve cervical symptoms (7, 9, 10). However, physiotherapy and manual therapy were not effective to improve dizziness symptoms (11).

Self-exercise consists of strength, mobilization, and oculomotor training and has been proposed as an add-on therapy to address both cervical and dizziness symptoms. One promising study evaluated the effectiveness of self-exercise for non-traumatic cervicogenic dizziness in seven patients and found an improvement in the patient's dizziness symptoms and range of motion (12).

To the best of our knowledge, this is the first randomized controlled trial to evaluate the efficacy of self-exercise as an add-on therapy in patients with non-traumatic cervicogenic dizziness.

## 2. Methods

### 2.1. Study design and setting

This randomized controlled trial was conducted from October 2018 to December 2020 at Khon Kaen University, Faculty of Medicine, Department of Otorhinolaryngology, Thailand.

### 2.2. Randomization

The randomization list was computer-generated by a statistician based on the block randomization method with randomly selected block sizes of 2, 4, 6, and 8. The allocation assignment was sealed in opaque, sequentially numbered envelopes.

### 2.3. Participants

We included all patients aged 18 years or older who had non-traumatic cervicogenic dizziness. The exclusion criteria were as follows: (1) the presence of trauma or recent surgery to the head, face, neck, or chest; (2) patients diagnosed with central vertigo; (3) those diagnosed with vertigo of vestibular origin such as benign positional paroxysmal vertigo; (4) patients who cannot perform self-exercise such as cervical fracture, and (5) those with history of allergy to dimenhydrinate or ibuprofen.

### 2.4. Diagnosis of non-traumatic cervicogenic dizziness

The modified stepwise process for diagnosing non-traumatic cervicogenic dizziness according to Reiley et al. (13) was used in this study.

Step 1. Patient history: The patient should have a history of neck pathology and also experience dizziness that has a close temporal relationship with the onset of cervical spine symptoms.
Step 2. Triage: Patients with traumatic cervical spine injury were excluded. Cervical arterial dysfunction was excluded when the patient reported dizziness during the 10-s active cervical range of motion test. The central vestibular disorders were excluded by a neurological screen including an assessment of radicular symptoms, myotomes, dermatomes, deep-tendon reflexes, upper motor neuron signs, and cranial nerve function.
Step 3. Vestibular assessment: The tests were done to exclude vestibular dysfunctions including evaluation of nystagmus, skew, smooth pursuit, saccades, the Dix-Hallpike test, static and dynamic visual acuity, and the head thrust test. In addition, a videonystagmography test was conducted to examine and rule out vestibular and central motor dysfunctions.
Step 4. Detailed cervical spine evaluation: A manual spinal examination was used to identify the source of pain. Patients with non-traumatic cervicogenic dizziness commonly present with tight posterior neck muscles and tenderness of both posterior neck muscles and cervical facet joints.

### 2.5. Procedure

**Figure 1:**
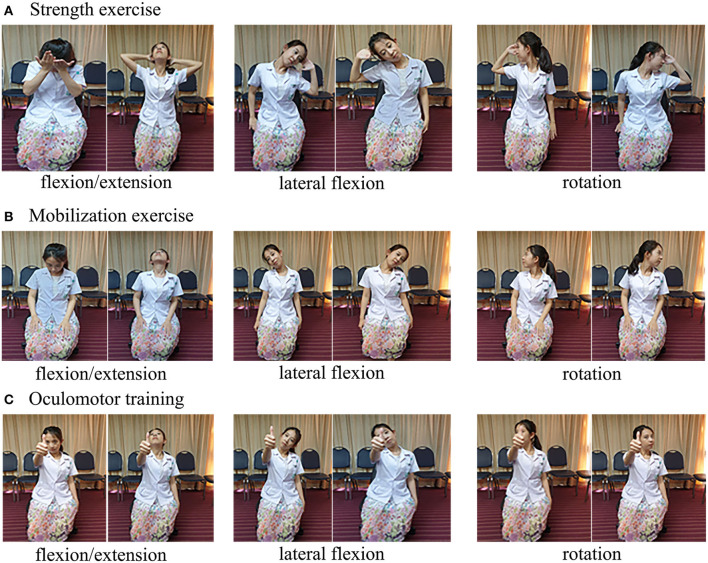
Self-exercise program. **(A)** Strength exercise. **(B)** Mobilization exercise. **(C)** Oculomotor training.

In the self-exercise group, the patients were instructed to perform the self-exercise at least 4 days per week and of 3 sets per day (12). The therapy includes the following factors (Figure 1):

1. Strength exercise
  a. Flexion: Put one hand on the front of the head and apply force in the same direction against the hand for 5 s × 10 times.
  b. Extension: Place the hands behind the head and perform opposing forces against the hands for 5 s × 10 times.
  c. Rotation: Put one of the hands on the side of the forehead and apply force in the direction to make a turn to the left or right of the head for 5 s × 10 times.
  d. Lateral flexion: Place the hand on the temporalis muscle and make the lateral flexion by isometric exercise, making opposing forces for 5 s × 10 times.
2. Mobilization exercise
  a. Flexion: Try to touch the chest with the chin. Hold for 5 s × 10 times.
  b. Extension: Slowly move the head backward and look upward. Hold for 5 s × 10 times.
  c. Rotation: Turn the head to the right and left while making a stop in the middle of the range. Hold for 5 s × 10 times.
  d. Lateral flexion: Tilt the head to the right and left making a stop in the middle of the range, keeping the shoulders down. Hold for 5 s × 10 times.
3. Oculomotor training
  a. Flexion: Set a fixed point in front of the eyes by holding out the arm and looking at the thumb. Try to touch the chest with the chin while keeping the eyes on the thumb for 5 s × 10 times.
  b. Extension: Set a fixed point in front of the eyes by holding out the arm and looking at the thumb. Slowly move the head backward and look upward while keeping the eyes on the thumb for 5 s × 10 times.
  c. Rotation: Set a fixed point in front of the eyes by holding out the arm and looking at the thumb. Turn the head to the right and left making a stop in the middle of the range while keeping the eyes on the thumb for 5 s × 10 times.
  d. Lateral flexion: Set a fixed point in front of the eyes by holding out the arm and looking at the thumb. Tilt the head to the right and the left, making a stop in the middle of the range, while keeping the eyes on the thumb for 5 s × 10 times.

There was no specific exercise instruction given to the control group. In the self-exercise group, all patients were instructed by the otolaryngologists until they were confident to practice the procedure at home.

### 2.6. Follow-up

The patients were followed up for 2 weeks.

### 2.7. Standard treatment procedure

The patients were advised to take dimenhydrinate (50 mg) when they had severe vertigo every 8 h and to stop taking it when the symptoms improved. All patients have also been prescribed ibuprofen 400 mg every 8 h to reduce cervical pain and to stop taking it when the symptoms improved.

### 2.8. Outcomes

#### 2.8.1. The Dizziness Handicap Inventory (DHI) questionnaire

The DHI questionnaire comprises 25 items to evaluate the self-perceived handicapping effects of dizziness symptoms. The patient was asked to answer each question as it pertains to dizziness or unsteadiness problems, specifically considering their condition during the last month. Questions were designed to incorporate the functional, physical, and emotional impacts on disability. Scores >10 points should be referred to balance specialists for further evaluation. The scores of 16–34, 36–52, and 54 points or more indicate mild, moderate, and severe handicaps (14). The DHI questionnaire has been validated as an assessment tool for non-traumatic cervicogenic dizziness (15).

#### 2.8.2. Neck Disability Index (NDI) questionnaire

The NDI questionnaire is designed to measure neck-specific disability. The questionnaire has 10 items concerning pain and activities of daily living including personal care, lifting, reading, headaches, concentration, work status, driving, sleeping, and recreation. Each item is scored out of five (with the no disability response given a score of zero) with a total score of 50 for the questionnaire. Higher scores represent greater disability (16).

#### 2.8.3. Visual analog scale (VAS) of neck pain

The VAS is a unidimensional measure of pain intensity, which has been widely used in diverse adult populations (17). The neck pain VAS is a continuous scale comprised of a horizontal line. The scale is anchored by “no pain” (score of 0) and “pain as bad as it could be” or “worst imaginable pain” (score of 10) (18).

#### 2.8.4. Cervical range of motion

The full cervical active range of motion was defined as follows: a flexion range of more than 50°, an extension range of more than 60°, a lateral flexion range of more than 45°, and a rotation range of more than 80° (19, 20).

#### 2.8.5. Posturography

Posturography was used to assess functional balance and relative contributions of visual, proprioceptive, and vestibular cues. Sensors within the platform footplate measure the force exerted by the feet when the patient's center of gravity is displaced (21).

We used the NeuroCom Balance Master, NeuroCom International, Inc., Oregon, USA to measure the sway velocity in this study. There was no standard normal value for the center of gravity sway velocity. However, from a study of 36 healthy subjects, the sway velocity when the eyes were open was 1.23° ± 0.14°/s (range 0.95–1.55) and when the eyes were closed was 2.15° ± 0.28°/s (range 1.70–2.85). The sway velocity will increase when changing the platform from a firm surface to a foam surface (22).

#### 2.8.6. Adverse events

The adverse events observed include falling, neck injury, and allergy.

### 2.9. Statistical analysis

The sample size was calculated base on previous study (12). The DHI score of the non-traumatic cervicogenic dizziness patients before treatment was 20.85 ± 11.53 points, and the DHI score after the treatment was 11.14 ± 7.28 points. With a significance level of 0.05 and a power of 90%, the total number of patients required was determined to be 32 patients.

Statistical analyses were performed using the SPSS version 20 and Stata version 14. Data were described as either means (for the continuous variables) or frequencies and percentages (for the categorical variables). Significant differences between groups were determined using the Student's *t*-test or the Mann–Whitney *U* test for continuous variables. The chi-square test or the Fisher's exact test was used to determine whether there was a significant difference between the expected frequencies and the observed frequencies. For all tests, a *p*-value of < 0.05 was considered statistically significant.

## 3. Results

**Figure 2:**
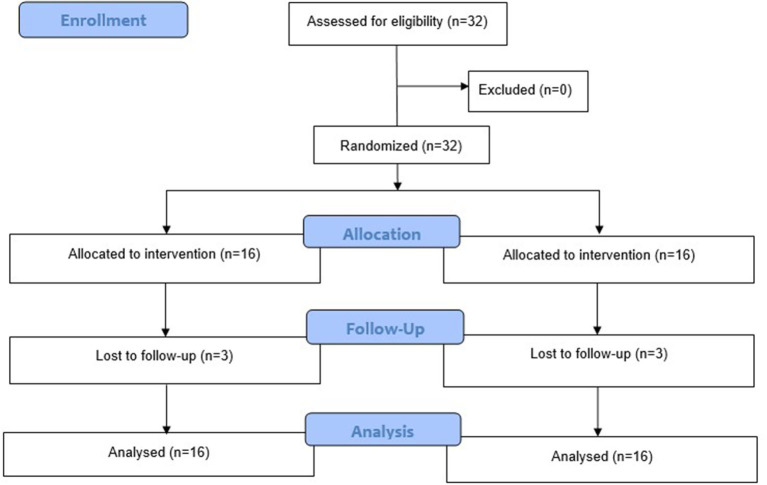
Participant flow diagram.

A total of 32 patients participated in this study, 16 of whom were allocated to the self-exercise group and 16 of whom were allocated to the control group. Three patients (18.75%) were lost to follow-up in both groups. The participant flow diagram is shown in Figure 2.

The average age of the participants was 48 years. Eight of the patients were men and 24 were women. There was no difference in sex, age, duration of vertigo, and underlying diseases (Table 1). At baseline, there was no difference in the DHI, the NDI, and the VAS of neck pain score between the groups (*p* > 0.05).

**Table 1:** Demographic data.

	**Self-exercise** ** (*n* = 16)**	**Control** ** (*n* = 16)**	***p*-value**
Gender (male:female)	3:13	5:11	0.41
Age (years)	47.5 ± 10.99	49.13 ± 9.65	0.66
Duration of dizziness (days)	59.94 ± 91.192	34 ± 36.06	0.30
**Underlying disease**			
-Hypertension	3	1	0.28
-Thyroid disease	3	0	0.28
-Breast mass	2	1	0.54
-Dyslipidemia	2	0	0.54
-Arrhythmia	1	2	0.54
-Anemia	1	0	0.99
-Benign prostatic hyperplasia	1	0	0.99
-Diabetes mellitus	1	0	0.99
-Endometriosis	1	0	0.99
-Hepatitis B virus infection	1	3	0.28
-Pituitary adenoma	1	0	0.99

After treatment, the DHI score was significantly lower in the self-exercise group [mean difference (MD) 25.92, 95% CI 4.21 to 47.63; lower is better]. The neck disability index score was also significantly lower in the self-exercise group (MD 6.16, 95% CI 0.42 to 11.88; lower is better). However, there was no difference in cervical pain score (MD 1, 95% CI −1.38 to 3.38; lower is better) (Table 2).

**Table 2:** DHI, NDI, and VAS score between groups.

	**Self-exercise (*n* = 16)**	**Control (*n* = 16)**	**Mean difference (95% CI)**	***p*-value**
**DHI**				
-Before	64.69 ± 22.23	55.38 ± 23.10	9.31 (−7.06 to 25.68)	0.254
-After	9.62 ± 9.84	35.54 ± 36.63	−25.92 (−47.63 to −4.21)	0.021^*^
**NDI**				
-Before	18.56 ± 7.12	14.81 ± 9.39	3.75 (−2.27 to 9.77)	0.213
-After	4.46 ± 3.71	10.62 ± 9.30	−6.16 (−11.88 to −0.42)	0.036^*^
**Cervical pain VAS**				
-Before	6.44 ± 2.63	6.75 ± 2.44	−0.31 (−2.14 to 1.52)	0.730
-After	3 ± 3.27	4 ± 2.58	−1 (−3.38 to 1.38)	0.395

^*^Statistically significance.

At baseline, four patients (25%) in the self-exercise group had a limited range of cervical motion and two patients (12.5%) in the control group had a limited range of cervical motion. After treatment, one patient (6.25%) in the self-exercise group had a limited range of cervical motion and one patient (6.25%) in the control group had a limited range of cervical motion. There was no statistically significant difference between the groups (Table 3).

**Table 3:** Range of motion results between groups.

**Limited range of motion**	**Self-exercise** ** (*n* = 16)**	**Control** ** (*n* = 16)**	***p*-value**
-Before	4 (25%)	2 (12.5%)	0.67
-After	1 (6.25%)	1 (6.25%)	

The mean sway velocity was decreased in both groups (lower is better). However, there was no significant difference between the groups (MD 0.08°/s, 95% CI −0.09 to 0.24, *p* = 0.35) (Table 4).

**Table 4:** Center of gravity sway velocity results between groups.

	**Self-exercise (n = 16)**	**Control (n = 16)**	**Mean difference (95% CI)**	***p*-value**
**Eyes open on a firm surface**				
-Before (°/s)	0.33 ± 0.25	0.22 ± 0.11	0.12 (−0.04 to 0.27)	0.139
-After (°/s)	0.32 ± 0.11	0.27 ± 0.12	0.05 (−0.05 to 0.14)	0.307
**Eyes closed on a firm surface**				
-Before (°/s)	0.44 ± 0.10	0.42 ± 0.22	0.02 (−0.12 to 0.15)	0.820
-After (°/s)	0.48 ± 0.17	0.37 ± 0.15	0.11 (−0.02 to 0.24)	0.103
**Eyes open on a foam surface**				
-Before (°/s)	0.55 ± 0.21	0.52 ± 0.18	0.03 (−0.14 to 0.18)	0.768
-After (°/s)	0.55 ± 0.19	0.50 ± 0.09	0.05 (−0.08 to 0.17)	0.437
**Eyes closed on a foam surface**				
-Before (°/s)	1.20 ± 0.40	1.25 ± 0.28	−0.05 (−0.33 to 0.23)	0.696
-After (°/s)	1.26 ± 0.66	1.15 ± 0.30	0.11 (−0.30 to 0.53)	0.572
**Composite score**				
-Before (°/s)	0.69 ± 0.20	0.60 ± 0.15	0.09 (−0.06 to 0.23)	0.242
-After (°/s)	0.65 ± 0.25	0.58 ± 0.14	0.08 (−0.09 to 0.24)	0.350

There was no difference in the number of standard treatment medication tablets used by the patients between the groups (Table 5). There were no major complications such as cervical spine injury or accidental falling, or allergy in both groups.

**Table 5:** Rescue medications.

	**Self-exercise (n = 16)**	**Control (n = 16)**	**Mean difference (95% CI)**	***p*-value**
Dimenhydrinate (tabs)	13.85 ± 8.70	13.92 ± 8.81	−0.07 (−7.16 to 7.01)	0.982
Ibuprofen (tabs)	6.97 ± 1.93	7.40 ± 2.05	−0.43 (−4.82 to 6.82)	0.726

## 4. Discussions

The perception of head rotation was from three afference systems including vestibular, proprioceptive, and visual receptors. Therefore, the abnormalities of these systems can induce a distorted sensation of head motion. The neck contains the neural system (afferent/efferent), the cardiovascular structures (carotid body and vertebral artery), and the cervico-proprioceptive system (cervical muscle and spine). Disorders of these systems can cause vertigo and dizziness (7, 23).

The treatment of non-traumatic cervicogenic dizziness with medications, such as NSAIDs and antihistamines, can reduce neck pain and vertigo symptoms. Physiotherapy and manual therapy were also proven to improve the range of motion (10, 24). However, they were not effective in improving dizziness symptoms (11).

We proposed that self-exercise consists of strength, mobilization, and oculomotor training as an add-on therapy that can help to relieve dizziness symptoms by improving balance and motor control.

In this study, we found that the dizziness handicap inventory score was significantly lower in the self-exercise group [mean difference (MD) 25.92, 95% CI 4.21 to 47.63; lower is better]. The neck disability index score was also significantly lower in the self-exercise group (MD 6.16, 95% CI 0.42 to 11.88; lower is better). However, there was no difference in cervical pain score (MD 1, 95% CI −1.38 to 3.38; lower is better) and mean sway velocity (MD 0.08°/s, 95% CI −0.09 to 0.24, *p* = 0.35).

The results from this study were consistent with those of Minguez-Zuazo et al. who examined the effectiveness of self-exercise in seven patients (12). They found an improvement in the range of motion, the dizziness handicap inventory score, and the neck disability index score.

Although the DHI was significantly improved, we found no difference in the center of gravity sway velocity. This lack of difference can be explained by the fact that, since the postural stability was not impaired before treatment, the improvement could only be small.

The strength of this study is the relatively large sample size when compared to other non-traumatic cervicogenic dizziness trials. To the best of our knowledge, this is the first randomized controlled trial to evaluate the efficacy of self-exercise as an add-on therapy for non-traumatic cervicogenic dizziness. The results of this study can be applied to help improve the quality of life of the patients.

In this study, we used the common assessment tools for dizziness, such as DHI, NDI, VAS, and posturography. There was a possibility to use an emerging tool to assess cervicogenic dizziness such as the Jenaer Standing Stability Score (25) or the Horizontal Plane Target Platform (26) in the future study.

The limitation of this study were a short follow-up period and the high use of rescue medication in both groups of patients, especially the usage of dimenhydrinate, which may affect the results. The recruitment period of this study was 2 years due to this condition being quite rare. We suggest that future studies should conduct as multicenter studies.

There was also a common limitation of this study and other cervicogenic dizziness trials due to a lack of standard diagnostic criteria for cervicogenic dizziness. We used the modified stepwise process to accurately rule in and rule out competing diagnoses (13). We propose that a cross-professional consensus on the diagnosis criteria of cervicogenic dizziness is needed.

## Conclusion

Self-exercise for non-traumatic cervicogenic dizziness is effective in reducing the dizziness symptom and its impact on daily life.

## Data availability statement

The original contributions presented in the study are included in the article/supplementary material, further inquiries can be directed to the corresponding author.

## Ethics statement

The studies involving human participants were reviewed and approved by Khon Kaen University Ethics Committee in Human Research (approval ID: HE611337). The patients/participants provided their written informed consent to participate in this study. Written informed consent was obtained from the individual(s) for the publication of any potentially identifiable images or data included in this article.

## Author contributions

PP contributed to study design, data acquisition, analysis and interpretation of results, statistical analysis, drafting of the manuscript, critical manuscript revision, and final approval. NT contributed to study design, data acquisition, statistical analysis, manuscript editing, and final approval. SS and KY contributed to study design, manuscript editing, and final approval. All authors contributed to the article and approved the submitted version.
